# Real‐world data of poly (ADP‐ribose) polymerase inhibitor response in Japanese patients with ovarian cancer

**DOI:** 10.1002/cam4.7149

**Published:** 2024-04-04

**Authors:** Ryosuke Uekusa, Akira Yokoi, Eri Watanabe, Kosuke Yoshida, Masato Yoshihara, Satoshi Tamauchi, Yusuke Shimizu, Yoshiki Ikeda, Nobuhisa Yoshikawa, Kaoru Niimi, Shiro Suzuki, Hiroaki Kajiyama

**Affiliations:** ^1^ Department of Obstetrics and Gynecology Nagoya University Graduate School of Medicine Nagoya Japan; ^2^ Institute for Advanced Research Nagoya University Nagoya Japan; ^3^ Department of Gynecologic Oncology Aichi Cancer Center Hospital Nagoya Japan

**Keywords:** niraparib, olaparib, ovarian cancer, PARP inhibitors, real‐world data

## Abstract

**Background:**

Poly (ADP‐ribose) polymerase (PARP) inhibitors have been increasingly used in the treatment of ovarian cancer, with *BRCA* positivity and homologous recombination deficiency (HRD) being common biomarkers used for predicting their efficacy. However, given the limitations of these biomarkers, new ones need to be explored.

**Methods:**

This retrospective study included 181 ovarian cancer patients who received olaparib or niraparib at two independent hospitals in Japan between May 2018 and December 2022. Clinical information and blood sampling data were collected. Patient characteristics, treatment history, and predictability of treatment duration based on blood data before treatment initiation were examined.

**Results:**

High‐grade serous carcinoma, *BRCA* positivity, HRD, and maintenance therapy after recurrence treatment were observed more frequently in the olaparib group than in the niraparib group. The most common reasons for treatment interruption were anemia, fatigue, and nausea in the olaparib group and thrombocytopenia in the niraparib group. Regarding response to olaparib treatment, complete response to the most recent treatment, maintenance therapy after the first chemotherapy, high‐grade serous carcinoma, and germline *BRCA* positivity were observed significantly more frequently among responders than among non‐responders. Furthermore, neutrophil counts were significantly higher among responders than among non‐responders.

**Conclusions:**

Inflammation‐related blood data, such as neutrophil count, obtained at the initial pre‐treatment visit might serve as potential predictors for prolonged olaparib treatment. While this study offers valuable insights into potential indicators for prolonged olaparib treatment, it underscores the need for more expansive research to strengthen our understanding of PARP inhibitors and optimize treatment strategies in ovarian cancer.

## 1 INTRODUCTION

Ovarian cancer is currently the third most common gynecologic malignancy and was the second leading cause of gynecologic cancer mortality globally in 2020.^1^ The cost of treating ovarian cancer per patient is among the highest of all cancer types. Although there have been investigations into early detection and prevention of ovarian cancer over the last decade, effective tools for general population screening are still lacking.^2^ Most ovarian malignancies are of epithelial origin, and the dualistic model divides ovarian cancer into two types: Type I cancer, such as mucinous, endometrioid, and clear cell carcinomas are slower‐growing, less aggressive tumors with specific genetic mutations, and Type II cancer, such as high‐grade serous carcinomas and undifferentiated carcinomas, are fast‐spreading, more aggressive tumors often with *p53* and *BRCA* gene mutations.^3^ High‐grade serous carcinoma, the most common subtype, is often detected in advanced stages and hence has a high mortality rate. The estimated 5‐year survival rate of this malignancy is approximately 30%. Despite an approximate 80% response rate to standard treatment including optimal debulking surgery and platinum‐based chemotherapy, most patients develop disease recurrence and progression within 2 years. Patients with ovarian cancer can experience multiple recurrences, which gradually develop into platinum resistance.^4^, ^5^ Therefore, prolonging the duration of progression‐free survival and augmenting the 5‐year survival rate are urgent challenges.

Poly (ADP‐ribose) polymerase (PARP) inhibitors represent a significant advancement in the treatment of advanced ovarian cancer in recent years.^6^ PARP is an essential enzyme for repairing single‐strand DNA breaks. The drug class known as PARP inhibitors functions by inhibiting PARP enzyme activity, leading to the accumulation of single‐strand breaks, which subsequently evolve into double‐strand breaks (DSBs). DSBs are mainly repaired through homologous recombination (HR) and nonhomologous end joining pathways.^7^ Cancer cells bearing *BRCA* mutations or homologous recombination deficiency (HRD) have an inherently impaired HRR pathway. Consequently, using PARP inhibitors to treat these cells causes further dysfunction in their DNA repair system by obstructing single‐strand breaks. This induces a state of synthetic lethality, wherein the concurrent effects of the dysfunctional HRR pathway and PARP inhibition cause excessive DNA damage, leading to selective cancer cell death.^8^

In Japan, olaparib was granted approval for multiple maintenance therapies, such as those for platinum‐sensitive recurrent ovarian cancer in 2018,^9^, ^10^ for *BRCA*‐mutated cases after remission from first‐line platinum chemotherapy in 2019,^11^ and for HRD cases in combination with bevacizumab after remission from first‐line platinum chemotherapy in 2020.^12^ Conversely, niraparib received approval as a maintenance treatment for platinum‐sensitive recurrent ovarian cancer,^13^ a maintenance treatment after remission from first‐line platinum chemotherapy,^14^ and as monotherapy for HRD and platinum‐sensitive recurrent ovarian cancer following the third or more chemotherapy sessions in 2020.^15^ Although rucaparib is approved for the treatment of ovarian cancer in the United States and Europe,^16^, ^17^ it is not available in Japan for the treatment of ovarian cancer.

The gold standard biomarker for PARP inhibitor efficacy has been HR repair defect status, including *BRCA* mutations, whereas its clinical biomarker has been platinum‐sensitive recurrent status. Studies have also shown that RAD51 foci,^18^ C‐terminal‐binding protein interacting protein,^19^ and *BRCA1* or *RAD51C* methylation^20^ can be used as biomarkers associated with homologous recombination repair. Recently, a report has suggested that [18F]FlourThanatrace uptake of positron emission tomography imaging predicts response to PARP inhibitors.^21^ However, none of them have been clinically applied.^22^ Only a few reports have investigated other clinical biomarkers for PARP inhibitors obtained from clinical data.^23^ Furthermore, there is growing interest in the use of real‐world data to address clinical questions unanswerable through clinical trial data.^24^, ^25^ Given that maintenance therapy follows initial treatment, the accumulation of clinical data takes time. Throughout Japan, olaparib and niraparib have been used for 5 and 2 years, respectively. Therefore, the current study aimed to examine the real‐world data for both drugs and determine whether available clinical data can be used to predict treatment duration.

## 2 MATERIALS AND METHODS

A retrospective analysis was conducted on data from 181 ovarian cancer patients who underwent olaparib and/or niraparib treatment at Nagoya University Hospital (Nagoya, Japan) and Aichi Cancer Center Hospital (Nagoya, Japan) from May 2018 to December 2022. Both the olaparib and niraparib groups consisted of patients who received maintenance therapy after first‐line platinum‐based chemotherapy for advanced epithelial ovarian, fallopian tube, or primary peritoneal cancer, and after platinum‐based chemotherapy for recurrence. Patient clinical information were comprehensively reviewed for age, body mass index (BMI), smoking and drinking habits, diabetes mellitus, histological type, g*BRCA* and HRD status, previous chemotherapy regimens, adverse effects, and blood sampling data at several points. This study was approved by the Ethics Committee of Nagoya University and the Ethics Committee of Aichi Cancer Center (Approval No. 2013‐0078). All procedures were performed in accordance with the relevant guidelines and regulations as well as with the requirements of the Declaration of Helsinki. Furthermore, informed consent was obtained from all participants.

Statistical analyses were conducted using GraphPad Prism 9 (GraphPad software). The Mann–Whitney *U*‐test and chi‐square test were utilized for comparisons between the two groups. A *p*‐value less than 0.05 was considered statistically significant.

## 3 RESULTS

### 3.1 Patient characteristics and treatment history

The olaparib and niraparib groups included 131 and 50 patients, had a median age of 59 (30–80) and 59 (23–80), and had a median BMI of 21.2 (14.2–32.8) and 21.9 (14.3–30.4), respectively (Table S1). No significant differences in smoking habits or diabetes incidence were observed between the two groups. However, a notably higher alcohol consumption was observed in the niraparib group than in the olaparib group (*p* = 0.01).

Regarding histological subtype, high‐grade serous carcinoma was observed significantly more frequently in the olaparib group (115/131, 87.8%) than in the niraparib group (32/50, 64.0%; *p* < 0.01). With respect to genetic profiles, germline *BRCA* mutation was observed in 26/131 (19.8%) and 2/50 (4.0%) patients in the olaparib and niraparib groups, respectively (*p* = 0.01). Meanwhile, HRD‐positive cases were observed in 19/131 (14.5%) and 4/50 (8.0%) patients in the olaparib and niraparib groups, respectively (*p* = 0.32).

Table S2 summarizes the treatment history of the patients. Accordingly, the median observation period was 697 (68–1699) and 423 (66–726) days in the olaparib and niraparib groups, respectively. The median treatment duration was 190 (14–1667) and 203 (5–726) days in the olaparib and niraparib groups, respectively. Treatment interruption due to adverse effects was observed in 22/131 (16.8%) and 6/50 (12.0%) patients in the olaparib and niraparib groups, respectively. Both groups exhibited comparable outcomes in response to their most recent treatment regimen.

Furthermore, 31/131 (23.7%) and 28/50 (56.0%) patients in the olaparib and the niraparib groups received maintenance therapy after first‐line chemotherapy, respectively. Meanwhile, 18/131 (13.7%) patients in the olaparib group received maintenance therapy in combination with bevacizumab. In addition, 5/131 (3.8%) and 10/50 (20.0%) patients in the olaparib and niraparib groups received maintenance therapy without surgery, respectively (*p* < 0.01). Treatment interruption was observed in 68/131 (51.9%) and 32/50 (64.0%) patients in the olaparib and niraparib groups, respectively (*p* = 0.87). The main reasons for treatment interruption were anemia (33/68, 48.5%) and fatigue and/or nausea (20/68, 29.4%) in the olaparib group and thrombocytopenia (16/32, 50.0%) in the niraparib group.

### 3.2 Relationship between blood data at initial pre‐treatment visit and duration of olaparib treatment

To evaluate the response to olaparib, responders were defined as those who continued treatment for at least 1 year including the interruption period, whereas non‐responders were defined as those whose treatment was terminated due to disease progression within 6 months. Among those in the olaparib group, 47 and 32 were responders and non‐responders, respectively, the characteristics of whom are detailed in Table 1. Responders and non‐responders had a median BMI of 22.2 (15.0–32.8) and 20.4 (15.9–29.5), respectively (*p* = 0.08). Complete response to the most resent treatment, maintenance therapy after first chemotherapy, serous carcinoma, and g*BRCA* positivity were observed significantly more frequently in responders than in non‐responders (*p* = 0.02, *p* = 0.04, *p* = 0.02, and *p* < 0.01, respectively). Conversely, the proportion of smokers was higher among non‐responders (*p* = 0.03).

**TABLE 1:** Characteristics of olaparib group patients divided by response.

	Responder (*N* = 47)	Non‐responder (*N* = 32)	*p*‐value
Age	59 (33–80)	57 (37–78)	0.84
BMI	22.3 (15.0–32.8)	20.4 (15.9–29.5)	0.08
Smoking	2 (4.3%)	7 (21.9%)	0.03
Drinking	1 (2.1%)	3 (9.4%)	0.30
DM	2 (4.3%)	4 (12.5%)	0.22
Number of previous chemotherapy regimens	2 (1–8)	2 (1–15)	
Response to most recent treatment			
CR	25 (53.2%)	8 (25.0%)	0.02
PR	22 (46.8%)	24 (75.0%)	
First maintenance	12 (25.5%)	2 (6.3%)	0.04
Treatment with Bev	5 (10.6%)	0 (0.0%)	0.08
Interruption	23 (48.9%)	10 (31.3%)	0.16
Histologic subtype			
Serous	46 (97.9%)	24 (75.0%)	0.02
Endometrioid	1 (2.1%)	4 (12.5%)	
Clear	0 (0.0%)	3 (9.4%)	
Unknown	0 (0.0%)	1 (3.1%)	
g*BRCA* mutation			
Positive	19 (40.4%)	0 (0.0%)	<0.01
Negative	12 (25.5%)	7 (21.9%)	
Unknown	16 (34.0%)	25 (78.1%)	
HRD			
Positive	7 (14.9%)	0 (0.0%)	NA
Negative	0 (0.0%)	0 (0.0%)	
Unknown	40 (85.1%)	32 (100%)	

Abbreviations: BMI, body mass index; CR, complete response; DM, diabetes mellitus; PR, partial response; HRD, homologous recombination deficiency.

**FIGURE 1:**
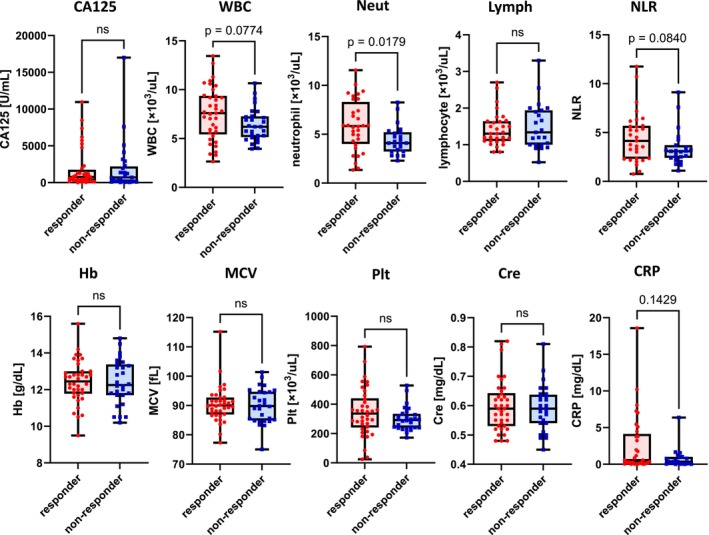
Comparison between responders and non‐responders in terms of blood data obtained at their first visit before treatment initiation in the olaparib group. Responders had significantly higher neutrophil counts (*p* = 0.02) and tended to have higher white blood cell counts, neutrophil–lymphocyte ratio (NLR), and C‐reactive protein (CRP) levels than did non‐responders. Lymph, lymphocyte; MCV, mean corpuscular volume; Neut, neutrophil; Plt, platelet; WBC, white blood cell.

We then compared blood samples from responders and non‐responders obtained at their initial visit before treatment initiation (Figure 1). Notably, responders had significantly higher neutrophil (*p* = 0.02) and tended to have higher white blood cell counts, neutrophil–lymphocyte ratio (NLR), and C‐reactive protein (CRP) levels than did non‐responders. A similar analysis in patients without g*BRCA* positivity showed that neutrophil counts were also significantly higher among responders than among non‐responders (Figure S1A; *p* = 0.04). Furthermore, a similar analysis in BRCA‐negative patients (Figure S1B) found no significant differences probably due to the small sample size, although neutrophil counts and CRP levels tended to be higher in responders than in non‐responders as well.

**FIGURE 2:**
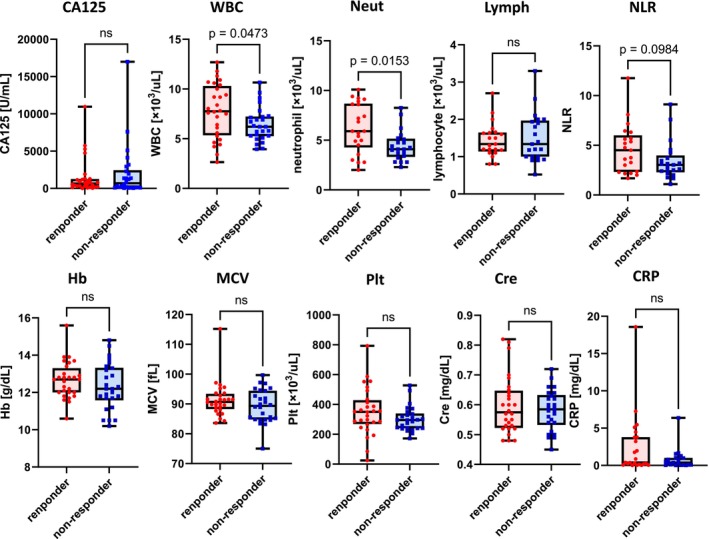
Comparison between responders and non‐responders in terms of blood data obtained at their first visit before treatment initiation only among patients in the olaparib group who received maintenance therapy after relapse. Notably, 35 and 30 patients were responders and non‐responders, respectively. White blood cell and neutrophil counts were significantly higher (*p* = 0.05, *p* = 0.02, respectively), whereas neutrophil–lymphocyte ratio (NLR), and C‐reactive protein (CRP) tended to be higher in responders than in non‐responders. Lymph, lymphocyte; MCV, mean corpuscular volume; Neut, neutrophil; Plt, platelet; WBC, white blood cell.

The same analysis in only patients who received maintenance therapy after relapse (Table S3, Figure 2 and Figure S2) revealed that in the overall population, white blood cell and neutrophil counts were significantly higher among responders than among non‐responders (*p* = 0.05 and 0.02, respectively). Among patients without g*BRCA* positivity, neutrophil counts tended to be higher in responders than in non‐responders (*p* = 0.11). Among g*BRCA*‐negative patients, no significant differences were found, although blood cell counts, neutrophil counts and CRP levels tended to be higher in responders than non‐responders as well.

In summary, these results suggest that patients with increased levels of inflammation‐related blood data at their initial pretreatment visit may receive a prolonged olaparib treatment course.

### 3.3 Relationship between blood data at the initial pre‐treatment visit and duration of niraparib treatment

Among those in the niraparib group, 9 and 12 were responders and non‐responders, respectively, the characteristics of whom are summarized in Table 2. Although no significant differences in any factors examined were observed in this population, complete response to most resent treatment tended to be higher among responders than among non‐responders (*p* = 0.08).

**TABLE 2:** Characteristics of niraparib group patients divided by response.

	Responder(*N* = 9)	Non‐responder(*N* = 12)	*p*‐value
Age	57 (30–76)	61 (52–73)	0.43
BMI	20.2 (18.2–28.2)	21.1 (18.6–30.4)	0.24
Smoking	2 (22.2%)	3 (25.0%)	>0.99
Drinking	3 (33.3%)	1 (8.3%)	0.27
DM	1 (11.1%)	2 (16.7%)	>0.99
Number of previous chemotherapy regimens	1 (1–3)	1 (1–3)	
Response to most recent treatment			
CR	7 (77.8%)	4 (33.3%)	0.08
PR	2 (22.2%)	8 (66.7%)	
First maintenance	7 (77.8%)	7 (58.3%)	0.64
Interruption	5 (55.6%)	5 (41.7%)	0.67
Histologic subtype			
Serous	4 (44.4%)	8 (66.7%)	0.52
Endometrioid	3 (33.3%)	1 (8.3%)	
Clear	1 (11.1%)	1 (8.3%)	
Carcinosarcoma	0 (0.0%)	2 (16.7%)	
Unknown	1 (11.1%)	0 (0.0%)	
*BRCA* mutation			
Positive	0 (0.0%)	1 (8.3%)	0.43
Negative	6 (66.7%)	5 (41.7%)	
Unknown	3 (33.3%)	6 (50.0%)	
HRD			
Positive	0 (0.0%)	0 (0.0%)	NA
Negative	1 (11.1%)	4 (33.3%)	
Unknown	8 (88.9%)	8 (66.7%)	

Abbreviations: BMI, body mass index; DM, diabetes mellitus; CR, complete response; PR, partial response; HRD, homologous recombination deficiency.

**FIGURE 3:**
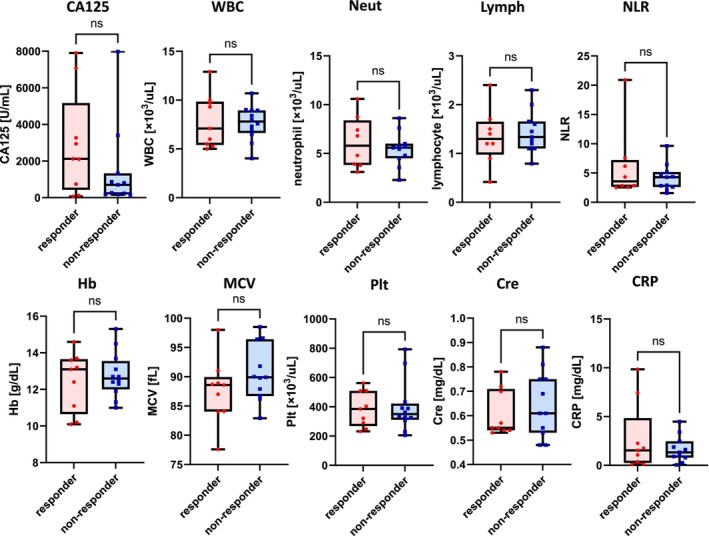
Comparison between responders and non‐responders in terms of blood data obtained at their first visit before treatment initiation in the niraparib group. No significant differences in any factors were observed. CRP, C‐reactive protein; Lymph, lymphocyte; MCV, mean corpuscular volume; Neut, neutrophil; NLR, neutrophil–lymphocyte ratio; Plt, platelet; WBC, white blood cell.

We then compared blood samples obtained from responders and non‐responders at their initial visit before treatment initiation (Table 2). Notably, no significant differences in any factors were observed between responders and non‐responders in the niraparib group (Figure 3). It was difficult to predict responses using blood samples obtained at the initial visit.

## 4 DISCUSSION

Ovarian cancer is characterized by high relapse rates and poor survival outcomes. Proteomics analysis provides a deeper understanding of the molecular mechanisms of cancer and technologies of proteomics, such as mass spectrometry and protein microarray, have advanced the understanding of biological processes, disease mechanisms, and the proteomic characterization of ovarian cancer. The advances can help the identification of new therapeutic targets, reduce drug resistance, and improve patient outcomes.^26^ Although previous treatment options had been limited to chemotherapy, the advent of PARP inhibitors has offered a promising direction in the therapeutic landscape for ovarian cancer.^27^ The phosphatidylinositol‐3 kinase (PI3K) pathway, which plays a crucial role in chemoresistance and the preservation of genomic stability, can be a target for novel therapeutic strategies and many treatments targeting the PI3K/AKT (protein kinase B)/mTOR pathways are being developed or are already in clinical studies.^28^ Besides, there have been reports that hyperthermic intraperitoneal chemotherapy (HIPEC) after cytoreductive surgery has improved the outcome of ovarian cancer, but it is not yet in the clinical practice stage.^29^

In this study, we aimed to explore real‐world data for olaparib and niraparib in Japan. While both agents act on similar biochemical pathways, their clinical applications diverge. Specifically, olaparib is predominantly utilized as maintenance monotherapy following first‐line chemotherapy for patients with *BRCA* mutations, as evidenced by the SOLO‐1 trial,^11^ and in combination with bevacizumab for HRD patients, as supported by the PAOLA‐1 trial.^12^ In contrast, the use of niraparib is not contingent on specific biomarker presence, as documented in the PRIMA trial.^14^ Therefore, a larger proportion of patients in the niraparib group would likely be on maintenance therapy after first‐line chemotherapy. In addition, *BRCA* mutations and HRD are frequently observed in high‐grade serous ovarian carcinoma (HGSOC),^30^ suggesting that the olaparib group may have had more patients with serous carcinoma, *BRCA*‐positive status, and HRD. Regarding adverse effects, anemia, fatigue, and nausea were the most common reasons for interruption in the olaparib group, whereas thrombocytopenia was the most common reason in the niraparib group, similar to that reported in prior studies.^9^, ^10^, ^11^, ^12^, ^13^, ^14^, ^15^

Although *BRCA* positivity and HRD are currently the gold standard biomarkers for PARP inhibitor efficacy, they are not without limitations. Therefore, this study sought to identify new biomarkers. Given that ovarian cancer can recur multiple times, minimally invasive biomarkers that do not rely on tissue information are important. Apart from *BRCA* positivity and HRD, a platinum‐free interval of >12 months and complete response after initial therapy have been cited as predictors of olaparib response^31^; however, no reports have been available on predictors of response based on data from the initial visit. The results of the current study suggest that patients with higher inflammation‐related values, such as white blood cell counts, neutrophil counts, and CRP levels, at diagnosis respond better to PARP inhibitors. Importantly, this has been the first study to show an association between inflammation values and olaparib efficacy based on the real‐world data. Despite being mainly known for its protective role in DNA repair, PARP‐1 also regulates inflammatory processes,^32^ and plays a key role in numerous biological processes. Accordingly, excessive PARP‐1 activity has been associated with several tumors and inflammation‐related clinical conditions, including asthma, sepsis, arthritis, atherosclerosis, and neurodegenerative diseases. Defects in DNA repair and chronic inflammation may both increase predisposition to cancer development. PARP inhibitors possess anti‐inflammatory activity and have been reported to have therapeutic potential in a wide range of inflammatory‐ and ischemia–reperfusion‐associated diseases, including cardiovascular diseases, diabetes, rheumatoid arthritis, endotoxic shock, and stroke.^33^, ^34^, ^35^, ^36^ Furthermore, HRD HGSOCs exhibit a range of DNA damage, interferon activation, and T cell inflammation.^37^ One explanation for the present results may be that the hyperinflammatory state of HGSOC could be associated with the anti‐inflammatory effects of PARP inhibitors. In addition, the use of PARP inhibitors leads to increased DNA damage in cancer cells, potentially resulting in a higher presentation of neoantigens. Tumors with HR deficiency tend to exhibit a greater neoantigen load compared to HR‐proficient tumors, possibly triggering a STING (stimulator of interferon genes)‐dependent innate immune response characterized by the induction of type I interferon and pro‐inflammatory cytokine production. Consequently, the combinations of PARP inhibitors with immunotherapies, such as CTLA‐4 and programmed death receptor‐1 (PD‐1)/PD‐L1 antibodies, could be an effective therapeutic strategy.^38^

The advantage of this study is that it is real‐world data on the largest number of Japanese people reported to date. This data is invaluable for understanding how these treatments are applied outside of clinical trial settings, including patient characteristics, treatment histories, and outcomes. In addition, this study showed the potential of blood sampling at the initial visit as a novel biomarker for response, which has not been reported before. This could lead to more personalized and effective treatment plans, as current biomarkers (*BRCA* positivity and HRD) have limitations. The current study also has several limitations. First, the sample size was small, especially in the niraparib group. This small sample size could reduce the statistical power of our findings, which suggests that the study might not have detected some significant differences despite being present. Moreover, the findings from a small sample might not be generalizable to the broader population of ovarian cancer patients. Furthermore, considering the fact that this work is a retrospective study, the results of this study require further investigation. Second, a large proportion of patients had unknown results for companion diagnoses such as *BRCA* and HRD. Without such information, it may be difficult to extract novel prognostic factors from patients who test negative for conventional biomarkers. Third, differences in patient backgrounds between the olaparib and niraparib groups made it difficult to compare their results. These differences can be a hindrance in ascertaining whether the observed outcomes were due to the treatments or were influenced by these background differences.

In conclusion, the current study examined real‐world data on olaparib and niraparib treatment, as well as treatment predictability based on blood data obtained before treatment initiation. Hematological parameters associated with inflammation, including neutrophil counts, obtained during the initial pre‐treatment consultation may function as potential prognostic markers for extended olaparib therapy. Although the present study provides meaningful contributions regarding potential markers for extended olaparib therapy, more comprehensive studies are essential to improve our understanding of PARP inhibitors and refine therapeutic approaches to ovarian cancer. Overall, this paper advances the understanding of PARP inhibitors in ovarian cancer treatment, proposes new directions for research, and contributes to the ongoing development of more personalized and effective therapeutic strategies.

## AUTHOR CONTRIBUTIONS

**Ryosuke Uekusa:** Conceptualization (equal); data curation (equal); formal analysis (lead); investigation (lead); methodology (equal); resources (equal); visualization (lead); writing – original draft (lead); writing – review and editing (lead). **Akira Yokoi:** Conceptualization (equal); data curation (equal); formal analysis (equal); investigation (equal); methodology (equal); resources (equal); supervision (lead); visualization (supporting); writing – original draft (supporting); writing – review and editing (supporting). **Eri Watanabe:** Conceptualization (equal); data curation (equal); formal analysis (equal); investigation (equal); methodology (equal); resources (equal); visualization (supporting). **Kosuke Yoshida:** Conceptualization (equal); investigation (equal). **Masato Yoshihara:** Conceptualization (equal); investigation (equal). **Satoshi Tamauchi:** Conceptualization (equal); investigation (equal). **Yusuke Shimizu:** Conceptualization (equal); investigation (equal). **Yoshiki Ikeda:** Conceptualization (equal); investigation (equal). **Nobuhisa Yoshikawa:** Conceptualization (equal); investigation (equal). **Kaoru Niimi:** Conceptualization (equal); investigation (equal). **SHIRO SUZUKI:** Conceptualization (equal); investigation (equal); supervision (supporting). **Hiroaki Kajiyama:** Conceptualization (equal); investigation (equal).

## FUNDING INFORMATION

This work was supported by JSPS KAKENHI Grant Number 21H03075 and the Princess Takamatsu Cancer Research Fund (No. 20‐25237). This study was also supported by the Program for Tokai Pathway to Global Excellence (T‐GEx) FY2021 and for Promoting the Enhancement of Research Universities as young researcher units for the advancement of new and undeveloped fields at Nagoya University.

## CONFLICT OF INTEREST STATEMENT

The authors have no conflict of interest.

## ETHICS STATEMENT

Approval of the research protocol by an Institutional Reviewer Board: This study was approved by the Ethics Committee of Nagoya University and the Ethics Committee of Aichi Cancer Center (Approval No. 2013‐0078).

## CONSENT

Informed consent was obtained from all participants.

## Supporting information


Data S1.

## Data Availability

The data that support the findings of this study are available from the corresponding author upon reasonable request.
