# Supplementary material for: Real‐world data of poly (ADP‐ribose) polymerase inhibitor response in Japanese patients with ovarian cancer
Source: Cancer Med. 2024 Apr 4;13(7):e7149. doi: 10.1002/cam4.7149 (PMC10993710; doi:10.1002/cam4.7149)

**Supplementary Table1. Patient characteristics**

|  |  | **Olaparib (N = 131)** | **Niraparib (N = 50)** | ***p* value** |
| --- | --- | --- | --- | --- |
| Age |  | 59 (30 - 80) | 59 (23 - 80) | 0.57 |
| BMI |  | 21.2 (14.2 - 32.8) | 21.9 (14.3 - 30.4) | 0.54 |
| Smoking |  | 14 (10.7 %) | 8 (16.0 %) | 0.59 |
| Drinking |  | 9 (6.9 %) | 10 (20.0 %) | 0.01 |
| DM |  | 9 (6.9 %) | 5 (10.0 %) | 0.53 |
| Histologic subtype |  |  |  |  |
|  | Serous | 115 (87.8 %) | 32 (64.0 %) | <0.01 |
|  | Endometrioid | 9 (6.9 %) | 6 (12.0 %) |  |
|  | Clear | 5 (3.8 %) | 5 (10.0 %) |  |
|  | Carcinosarcoma | 0 (0.0 %) | 1 (2.0 %) |  |
|  | Unknown | 2(1.5 %) | 6 (12.0 %) |  |
| *BRCA* status |  |  |  |  |
|  | Positive | 26 (19.8 %) | 2 (4.0 %) | 0.01 |
|  | Negative | 36 (27.5 %) | 26 (52. 0%) |  |
|  | Unknown | 69 (52.7 %) | 22 (44. 0%) |  |
| HRD |  |  |  |  |
|  | Positive | 19 (14.5 %) | 4 (8.0 %) | 0.32 |
|  | Negative | 2 (1.5 %) | 12 (24.0 %) |  |
|  | Unknown | 110 (83.9 %) | 33 (66.0 %) |  |

BMI, body mass index; DM, diabetes mellitus; HRD, homologous recombination deficiency

**Supplementary Table2. Treatment history**

|  | | **Olaparib (N = 131)** | **Niraparib (N = 50)** | ***p* value** |
| --- | --- | --- | --- | --- |
| Observation days (days) | | 697 (68 - 1699) | 423 (66 - 726) |  |
| Duration to the treatment (days) | | 190 (14 - 1667) | 203 (5 - 726) |  |
| Reason for termination | |  |  |  |
|  | PD | 63 (48.1 %) | 29 (58.0 %) |  |
|  | Adverse effect | 22 (16.8 %) | 6 (12.0 %) |  |
|  | Others | 2 (0.0 %) | 1 (2.0 %) |  |
| Number of previous chemotherapy regimens | |  |  |  |
|  | 1~4 | 114 (87.0 %) | 49 (98.0 %) |  |
|  | 5~9 | 13 (9.9 %) | 1 (2.0 %) |  |
|  | 10~ | 4 (3.1 %) | 0 (0.0 %) |  |
| Response to most recent treatment | |  |  |  |
|  | CR | 62 (47.3 %) | 27 (54.0 %) | 0.51 |
|  | PR | 69 (52.7 %) | 23 (46.0 %) |  |
| Maintenance for first-line chemotherapy | | 31 (23.7 %) | 28 (56.0 %) | < 0.01 |
| Treatment with Bev | | 18 (13.7 %) | - |  |
| No surgery | | 5 (3.8 %) | 10 (20.0 %) | < 0.01 |
| Interruption | | 68 (51.9 %) | 32 (64.0 %) | 0.87 |
| Reason for interruption | |  |  |  |
|  | Anemia | 33 (48.5 %) | 8 (25.0 %) |  |
|  | Neutropenia | 17 (25.0 %) | 5 (15.6 %) |  |
|  | Thrombocytopenia | 6 (8.8 %) | 16 (50.0 %) |  |
|  | Fatigue | 14 (20.6 %) | 2 (6.3 %) |  |
|  | Nausea | 11 (16.2 %) | 0 (0.0 %) |  |
|  | Others | 8 (11.8 %) | 7 (21.9 %) |  |

CR, complete response; PR, partial response; PD, progressive disease

**Supplementary table3. Characteristics of olaparib group patients who received maintenance therapy after relapse divided by response**

BMI, body mass index; DM, diabetes mellitus; CR, complete response; PR, partial response; Bev, bevacizumab; HRD, homologous recombination deficiency

|  | | Responder (N=35) | Non-responder (N=30) | p value |
| --- | --- | --- | --- | --- |
| Age | | 60 (37-80) | 55 (37-76) | 0.25 |
| BMI | | 22.8 (15.0-32.8) | 20.4 (15.9-29.5) | 0.08 |
| Smoking | | 2 (5.7%) | 6 (20.0%) | 0.13 |
| Drinking | | 1 (2.9%) | 2 (6.7%) | 0.59 |
| DM | | 2 (5.7%) | 4 (13.3%) | 0.40 |
| Number of previous chemotherapy regimens | | 2 (1-8) | 3 (2-15) | 0.02 |
| Response to most recent treatment | |  |  |  |
|  | CR | 14 (40.0%) | 6 (20.0%) | 0.11 |
|  | PR | 21 (60.0%) | 24 (80.0%) |  |
| Treatment with Bev | | 5 (14.3%) | 0 (0.0%) | 0.06 |
| Interruption | | 18 (51.4%) | 4 (13.3%) | <0.01 |
| Histologic subtype | |  |  |  |
|  | Serous | 34 (97.1%) | 24 (75.0%) | <0.01 |
|  | Endometrioid | 1 (2.9%) | 4 (12.5%) |  |
|  | Clear | 0 (0.0%) | 3 (9.4%) |  |
|  | Mucinous | 0 (0.0%) | 1 (3.1%) |  |
| *BRCA* mutation | |  |  |  |
|  | Positive | 19 (40.4%) | 0 (0.0%) | <0.01 |
|  | Negative | 12 (25.5%) | 7 (21.9%) |  |
|  | Unknown | 16 (34.0%) | 25 (78.1%) |  |
| HRD | |  |  |  |
|  | Positive | 7 (14.9%) | 0 (0.0%) | >0.99 |
|  | Negative | 0 (0.0%) | 0 (0.0%) |  |
|  | Unknown | 40 (85.1%) | 32 (100%) |  |

**Supplementary figure1.**


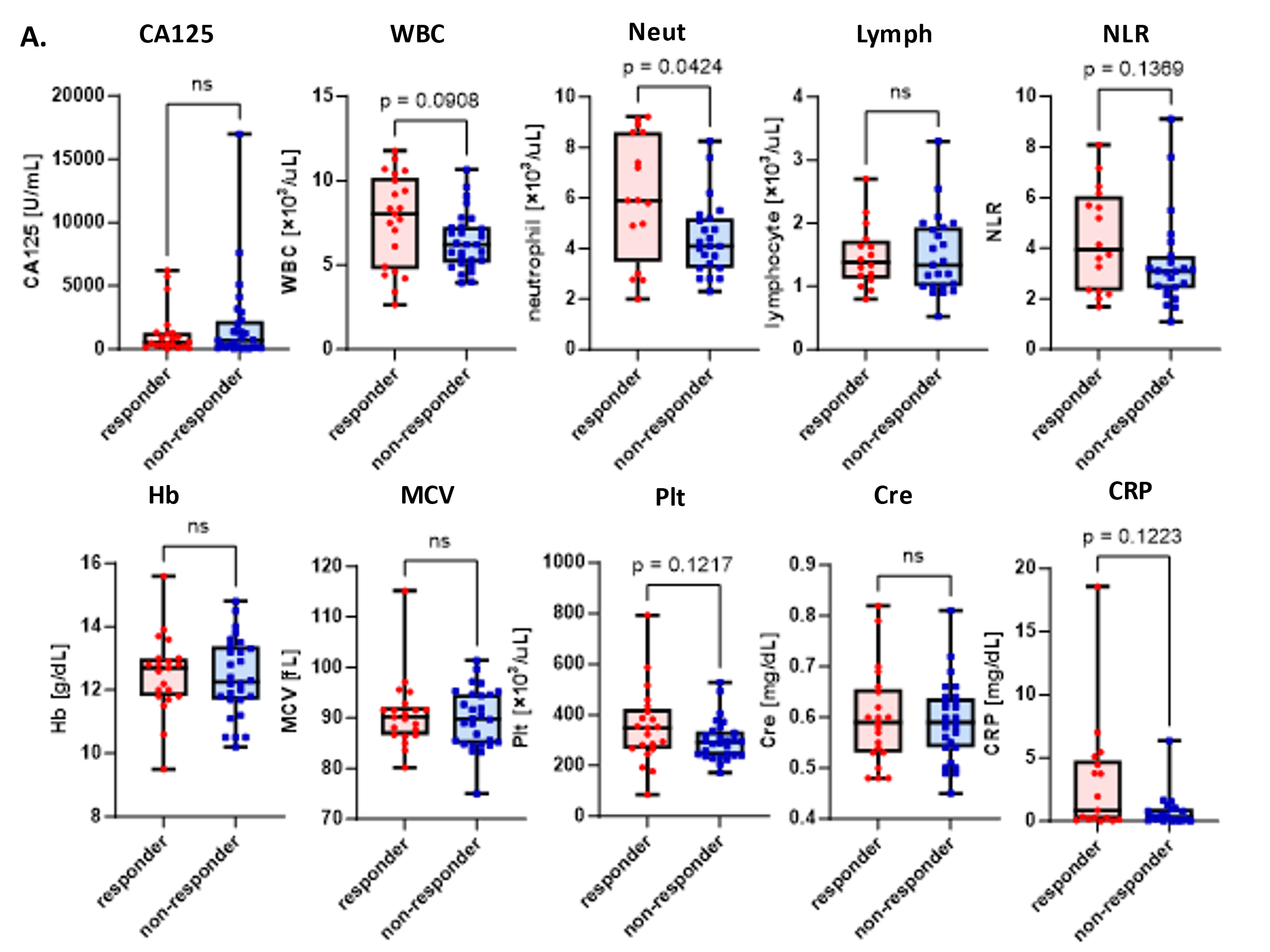


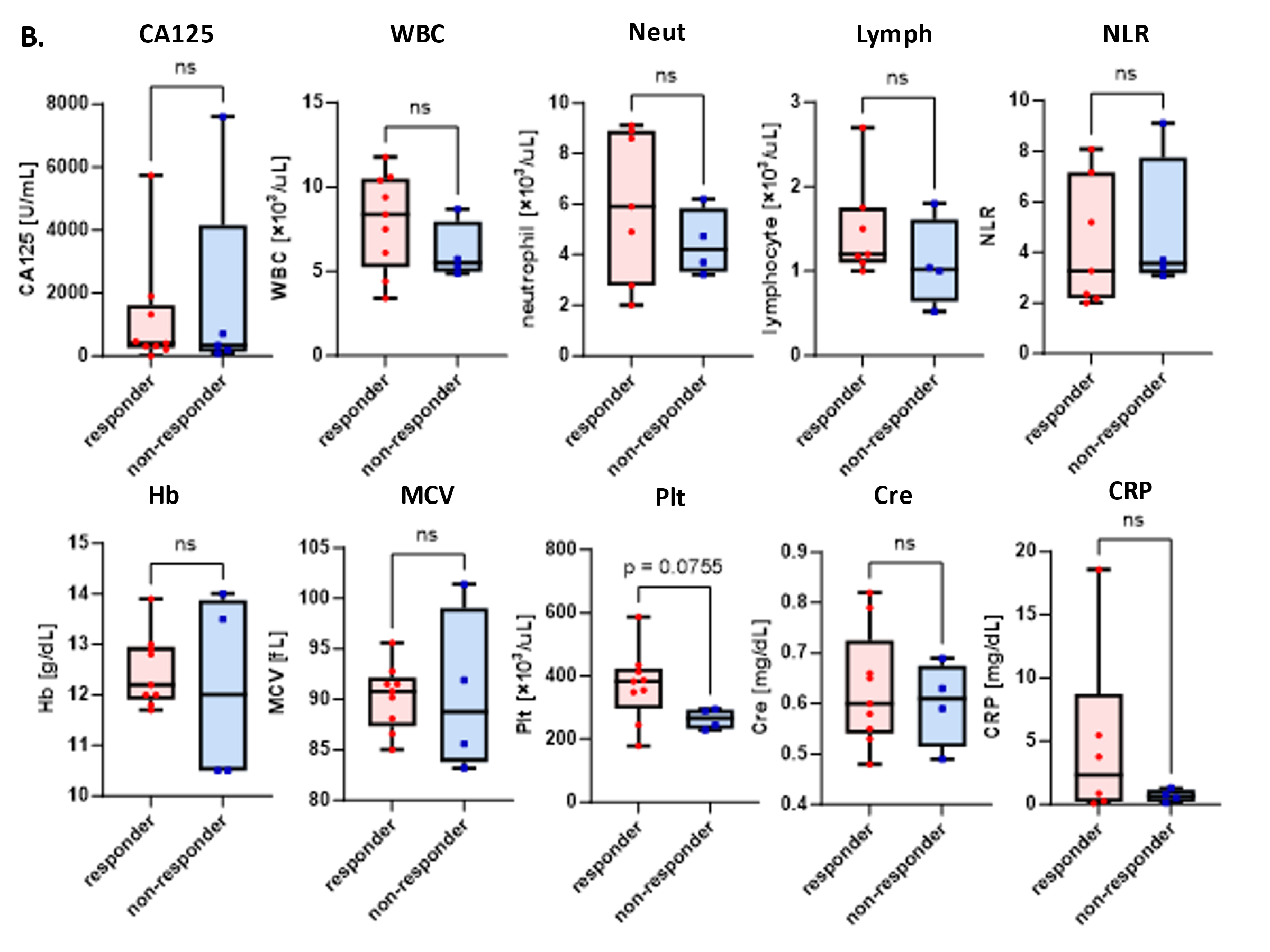


**Supplementary figure2.**


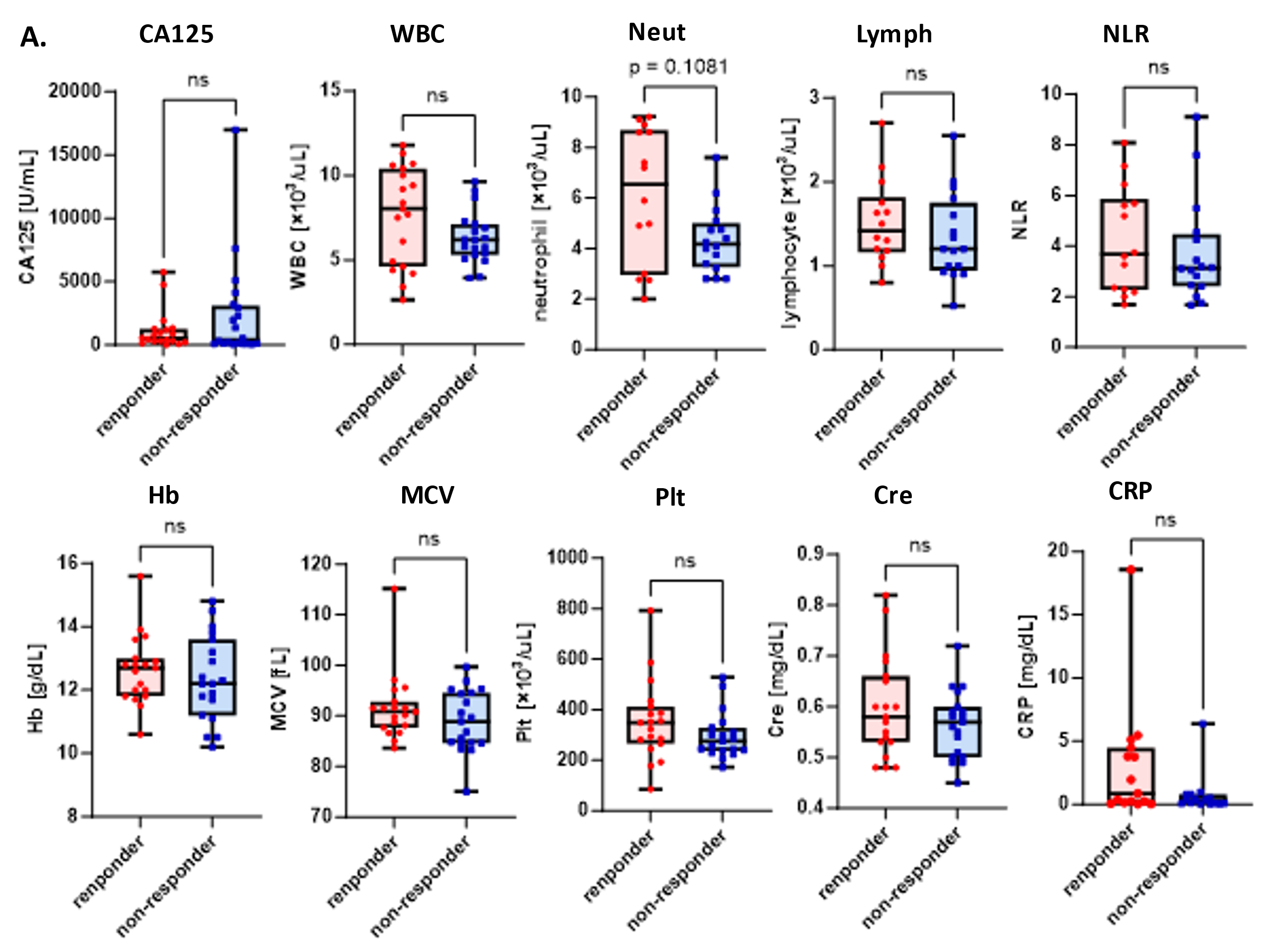


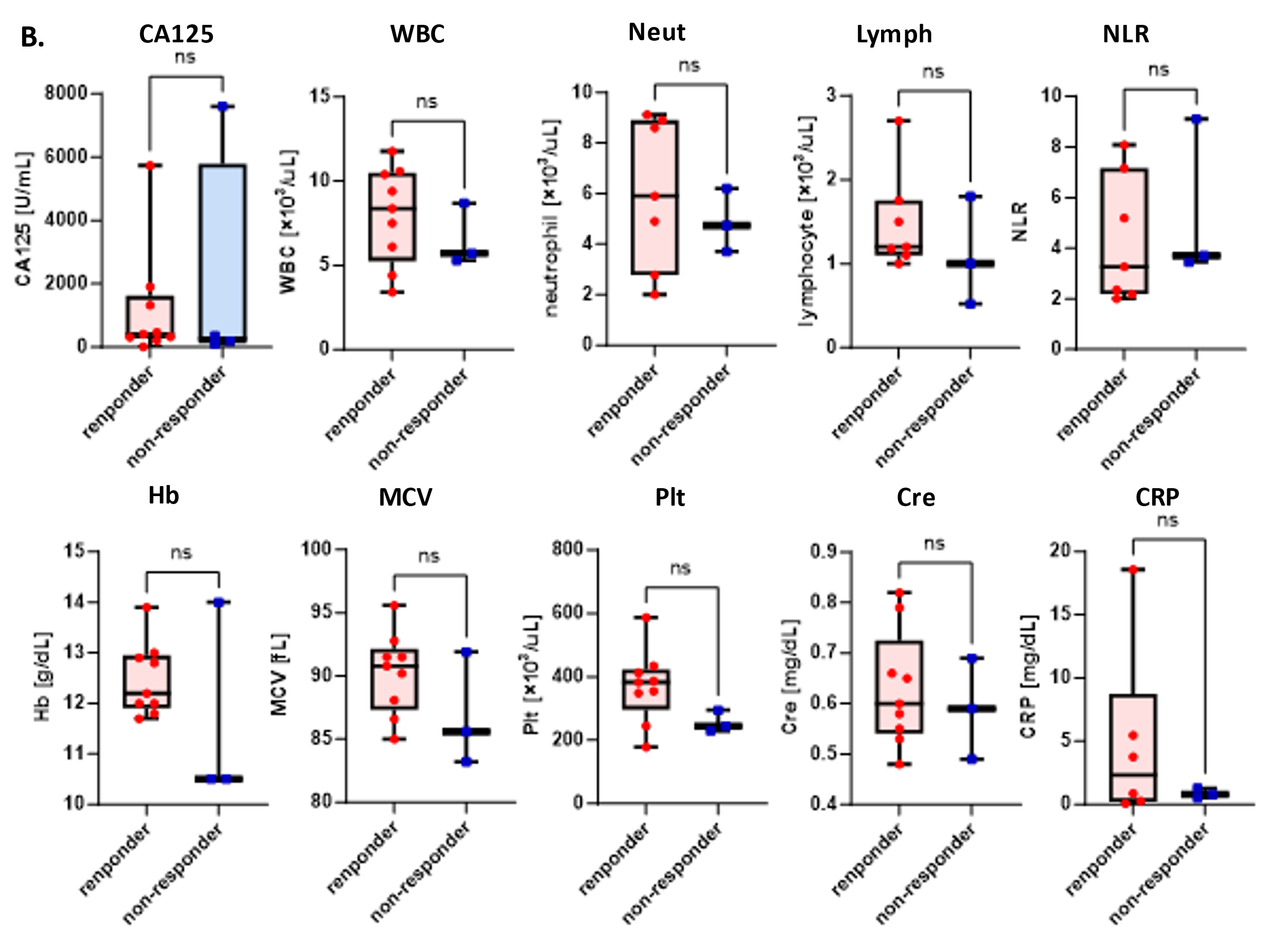

Supplement: Supplementary file 1 — Data S1. [file CAM4-13-e7149-s001.docx]
